# A Thermocycler Using a Chip Resistor Heater and a Glass Microchip for a Portable and Rapid Microchip-Based PCR Device

**DOI:** 10.3390/mi13020339

**Published:** 2022-02-21

**Authors:** Dongsun Yeom, Jeongtae Kim, Sungil Kim, Sanghoon Ahn, Jiyeon Choi, Youngwook Kim, Chiwan Koo

**Affiliations:** 1 Department of Electronic Engineering, Hanbat National University, Daejeon 34158, Korea; yds1410@naver.com (D.Y.); jeotae@daum.net (J.K.); 2 Department of Laser and Electron Beam Application, Korea Institute of Machinery and Materials, Daejeon 34103, Korea; sung1@kimm.re.kr (S.K.); shahn@kimm.re.kr (S.A.); jchoi@kimm.re.kr (J.C.); 3 Department of Electronic Engineering, Sogang University, Seoul 04107, Korea; youngkim@sogang.ac.kr

**Keywords:** polymerase chain reaction, glass PCR microchip, chip resistor heater, SLE

## Abstract

This study proposes a rapid and inexpensive thermocycler that enables rapid heating of samples using a thin glass chip and a cheap chip resistor to overcome the on-site diagnostic limitations of polymerase chain reaction (PCR). Microchip PCR devices have emerged to miniaturize conventional PCR systems and reduce operation time and cost. In general, PCR microchips require a thin-film heater fabricated through a semiconductor process, which is a complicated process, resulting in high costs. Therefore, this investigation substituted a general chip resistor for a thin-film heater. The proposed thermocycler consists of a compact glass microchip of 12.5 mm × 12.5 mm × 2 mm that could hold a 2 μL PCR sample and a surface-mounted chip resistor of 6432 size (6.4 mm × 3.2 mm). Improving heat transfer from the chip resistor heater to the PCR reaction chamber in the microchip was accomplished via the design and fabrication of a three-dimensional chip structure using selective laser-induced etching, a rapid prototyping technique that allowed to be embedded. The fabricated PCR microchip was combined with a thermistor temperature sensor, a blower fan, and a microcontroller. The assembled thermocycler could heat the sample at a maximum rate of 28.8 °C/s per second. When compared with a commercially available PCR apparatus running the same PCR protocol, the total PCR operating time with a DNA sample was reduced by about 20%.

## 1. Introduction

Polymerase chain reaction (PCR) is an indispensable technology that is used to amplify deoxyribonucleic acid (DNA) in various fields such as molecular biology, medical science, criminal investigations, and disease diagnosis because of its high selectivity and high sensitivity [1,2,3]. Especially, this technology in the disease diagnosis has the advantage of detecting a very small amount of target (viruses or bacteria) in the body for early diagnosis. Therefore, the literature contains several reports of several point-of-care (POC) PCR devices being used to conduct on-site testing in locations with large floating populations such as airports or ports to prevent the spread of highly infectious diseases [4,5,6,7,8]. In the typical PCR process, the thermal cycle of 94 °C–60 °C–72 °C is repeated 30 to 40 times. This process involves the low heating rate of a conventional thermal cycler in heating and cooling tens of microliter volume PCR reaction samples, with a duration of 2 to 3 h, an extended period that poses an obstacle in POC diagnosis [9,10].

Recent advances in microfluidics and micro/nano fabrication technologies led to the development of various miniaturized PCR systems. These systems used a microchip requiring only a few micro- to nano-liter volume samples [11,12,13,14,15]. In addition, various heating methods were reported to reduce heating and cooling time in miniaturized PCR systems. In general, microchips were heated by a thin-film metal heater, a thermoelectric module (Peltier element), a cement resistor, or other methods.

When using a metallic thin-film heater that is patterned on the surface of a microchip, the distance between the heater and the reaction chamber in the microchip is small to facilitate the rapid heating of the reaction sample due to excellent heat conduction. Lim et al. presented a platinum (Pt) thin-film on a quartz chip, serving as a heater and a temperature sensor. The PCR microchip, composed of polyethylene terephthalate (PET) and polyimide (PI), was placed between heater chips [16]. Koo et al. reported a microchip fabrication by bonding a heater-patterned glass chip using gold (Au) and a chamber-etched glass microchip, which heated a sample at a maximum rate of 5.5 °C per second [17]. Veltkamp et al. similarly used Au as a heater and temperature sensor on a glass PCR chip [18]. These PCR microchips with thin-film metal heaters provide good heating efficiency. However, fabricating these chips can be costly due to the inherent complexity of using microelectronic mechanical Systems (MEMS) processes such as metal deposition, photolithography, and etching. Recently, printed circuit board (PCB)-based heater devices using thin metal lines on a PCB as a heater have been developed. For example, Kaprou et al. reported a PCB-based heater combined with a microfluidic channel to conduct continuous flow and static PCR methods [19,20].

Gou et al. and Sreejith et al. reported PCR systems using a Peltier heater. This technique used a single element to heat and cool and provided high reliability in terms of temperature control. However, the device was expensive to manufacture and exhibited high power consumption compared to other external heaters. In addition, a Peltier heater needs to incorporate a cooling system for effective heating. For this reason, this approach required additional components such as a heatsink and cooling fan, which increased the size of the system [21,22].

Mendoza-Gallegos et al. reported a PCR system using inexpensive cement resistors. However, the non-uniform surface temperature of a cement resistor may lead to an imbalance in the PCR sample temperature [23].

We propose a low-cost, compact commercial surface-mount device (SMD) chip resistor-based heater to overcome these limitations. A chip resistor heater can generate rapid heating by using an overcurrent, and its compact size allows localized heating of the small reaction chamber of the microchip. Moreover, the chip resistor heater device requires no additional fabrication since standard chip resistors are commercially available, and the flat surface allows simple placement on a microchip. We improved heat transfer from the chip resistor heater to the PCR reaction chamber in the proposed microchip by simulating and fabricating a three-dimensional chip structure in which the chip resistor could be embedded. A blower fan and a temperature sensor were combined with the chip resistor-based heater. A microcontroller controlled the temperature of the PCR reaction chamber with proportional–integral–derivative (PID) feedback. A black plastic housing designed to enclose all components was fabricated using a fused deposition modeling (FDM)-type 3D printer. The fabricated chip resistor-based heater was demonstrated by amplifying DNA (ZFP36, Human tagged ORF clone) and conducting gel-electrophoresis.

As a material commonly used in PCR microchips, glass was selected for this device. Polymers and glass are widely used because they are biocompatible and optically transparent. Typically, polydimethylsiloxane (PDMS), polymethyl methacrylate (PMMA), and polycarbonate (PC) are used in low-cost polymer microchips. However, PDMS has gas permeability and difficulty in mass production. PMMA’s low glass transition temperature of 105 °C makes it unsuitable for the PCR denaturation step at high temperatures (about 95 °C). PC has a disadvantage in optical detection in real-time microchip PCR systems because of its autofluorescence [24,25]. In contrast, glass has high light transmittance, heat resistance, electrical insulation, and high thermal conductivity compared to polymer [26]. Glass has its own limitations, of course, including the long fabrication time and effort required for micromachining, along with the need for special tools or a clean room facility. Using a two-step selective laser-induced etching (SLE) process, these limitations were overcome. The first step of the process involves modifying the target area of the glass chip using the nonlinear absorption of a femtosecond laser. Next, wet etching is used to remove the portion modified by the laser. This process allows the fabrication of a 3D hollow structure inside one single glass without a bonding process, making it useful for rapidly fabricating a prototype glass microchip [27,28,29]. Earlier, we reported an optimization method for fabricating 3D structures in a single glass sheet using the SLE process [30,31]. This study used the optimized SLE process conditions to fabricate a PCR reaction chamber and a chip resistor slot in the glass microchip.

## 2. Materials and Methods

**Figure 1:**
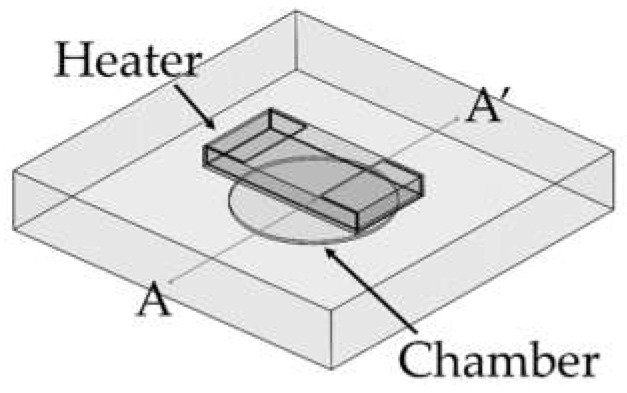
Illustration of the PCR microchip using a chip resistor as a heater. The chamber was located in a glass substrate (12.5 mm × 12.5 mm × 2 mm), and the diameter and the height of the PCR reaction chamber were 5 mm and 0.1 mm, respectively.

A PCR microchip with a chip resistor heater was designed, and finite element method (FEM) software was used to simulate the temperature profile of the microchip when heated by the chip resistor heater. As shown in Figure 1, a circular PCR reaction chamber was placed inside a glass substrate, and the heater was placed above the chamber. The dimension of the glass substrate was 12.7 mm × 12.7 mm × 2 mm, small enough to be placed in a portable thermocycler. The diameter of the chamber was 5 mm, and the height was 0.1 mm, in order to hold 2 μL of PCR sample to be amplified. The temperature profile around the chamber was simulated while moving the position of the heater. Three distances between the heater and the chamber (600 μm, 950 μm, and 1300 μm) were used for the simulation. The chamber’s inlet and outlet were not considered in the simulation.

**Figure 2:**
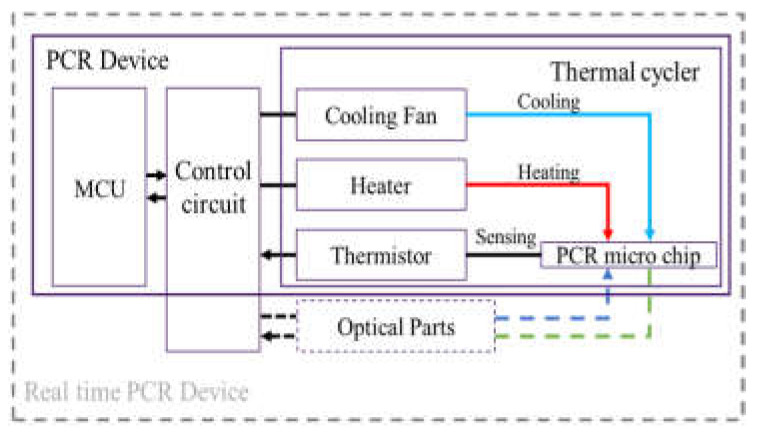
An overview of the PCR device, including a thermal cycler, control circuit, and microcontroller unit (MCU).

We fabricated a portable PCR device to amplify DNA samples to demonstrate the proposed combination of a microchip and chip resistor heater. Figure 2 illustrates the PCR device, consisting of a thermocycler, a control circuit, and a microcontroller unit (MCU). The thermocycler comprises a microchip, a chip resistor heater, a blower fan, and a thermistor. The control circuit includes peripheral circuits for switching current, and the MCU controls the PCR thermocycling temperature. In addition, a hole is included at the bottom of the thermocycler to allow the addition of an optical detection component to the thermocycler for expansion into a real-time PCR device.

**Figure 3:**
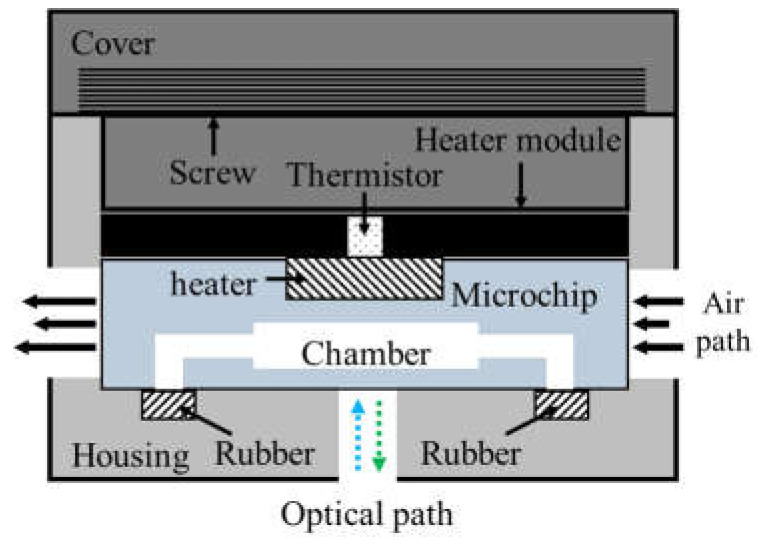
Schematic of the thermocycler module.

As shown in Figure 3, the thermocycler module consisted of a heater module, a microchip, rubber, and a housing with a cover. The heater module included the chip resistor heater and the thermistor for heating the microchip and sensing the temperature of the microchip. A blower fan, used to accelerate the cooling of the microchip, rotated at 3400 rpm and blew the hot air in the thermocycler module to the outside. The microchip’s inlet and outlet were sealed with rubber. In addition, a screw structure was manufactured on the cover and the housing. Rotating the cover to close the housing caused the rubber, the microchip, and the heater module to be pressed together with strong pressure, which prevented PCR sample evaporation and improved heat transfer between the chip resistor and the microchip.

**Figure 4:**
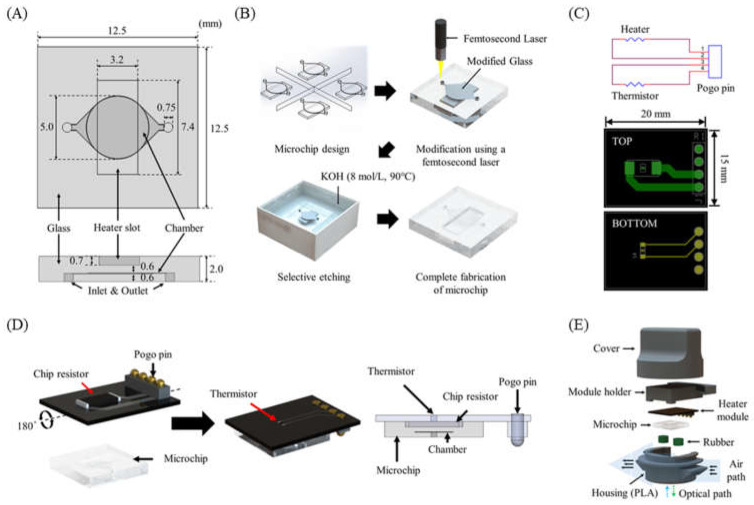
(**A**) PCR microchip. (**B**) Microchip fabrication steps using the SLE process. (**C**) Heater module circuit and PCB design. (**D**) Schematic of the heater module including a chip resistor heater and a thermistor, and the integrated heater module with the microchip. (**E**) Components of the thermocycler module.

The glass PCR microchip was fabricated by the SLE process using a femtosecond laser according to the design determined through the temperature profile simulation. The distance between the chip resistor heater and the PCR reaction chamber was 600 μm. In addition, a heater slot was designed so that the chip resistor heater could be placed at the exact center of the PCR reaction chamber (Figure 4A). To fabricate cavity structures for the PCR chamber and the heater slot in the glass microchip, we performed a two-step process with laser direct writing and etching with the optimized SLE process conditions (Figure 4B) [30,31]. This technique allowed the fast and easy fabrication of a 3D cavity inside the glass.

As shown in Figure 4C, a single PCB (15 mm × 20 mm × 0.7 mm) for the heater module was designed. The chip resistor heater and the thermistor were placed on each side of the PCB to allow the thermistor to directly measure the temperature of the chip resistor heater. A POGO pin was used for a simple electrical connection to the control circuit and easy replacement of the heater module (Figure 4D). The heater module was integrated with the microchip by placing the chip resistor heater into the slot of the microchip.

Rubber septa were used to seal the PCR microchip’s inlet and outlet and prevent evaporation during PCR. The housing and the cover were designed to enclose all components of the thermocycler (Figure 4E) and prototyped with an FDM-type 3D printer and black polylactic acid (PLA) filament.

Next, the thermocycler module was connected to the control circuit composed of two metal–oxide–semiconductor field-effect transistors (MOSFET) and a voltage divider. The MOSFETs switched electrical current to the chip resistor heater and the blower fan, while the voltage divider circuit was used to measure the temperature of the thermistor. The MCU provided thermocycling by simultaneously controlling the MOSFET switches and collecting temperature data. In the MCU, the PID control was processed by calculating the error from the target temperature value by receiving feedback about the current temperature of the sample [32]. An Arduino was used as the MCU to connect with many peripheral devices.

To verify the DNA amplification of our PCR device, we employed a commercial PCR device (PCR Thermal Cycler Dice^®^ Gradient, TaKaRaBio, Japan, Heating rate: 3.0 °C/s, Cooling rate: 2.0 °C/s). The templated DNA of Human Tagged ORF Clone (ZFP36, ORIGENE, Rockville, MD, USA) was used to compare our PCR device with the commercial PCR device. The primer gene consisted of a forward primer (5′ ATT AGG ACA AGG CTG GG 3′) and a reverse primer (5′ GGA CTT AAA ATG TCG 3′) to produce a 978 bp amplicon. Bovine serum albumin (BSA) was dissolved in phosphate-buffered saline (PBS) at a ratio of 2 μg/μL to prepare a BSA solution for preventing DNA adsorption on the surface of the reaction chamber in the PCR microchip. The BSA solution was loaded into the reaction chamber for 60 min at room temperature. Then, the chamber was rinsed with PBS and a PCR mix sample was loaded into the reaction chamber. The composition ratio of the PCR sample is listed in Table 1. PCR was carried out under the following conditions: 30 s at 95 °C (pre-denaturation), followed by 30 cycles of 95 °C for 30 s (denaturation), 30 s at 60 °C (annealing), and 30 s at 72 °C (extension).

## 3. Results & Discussion

**Figure 5:**
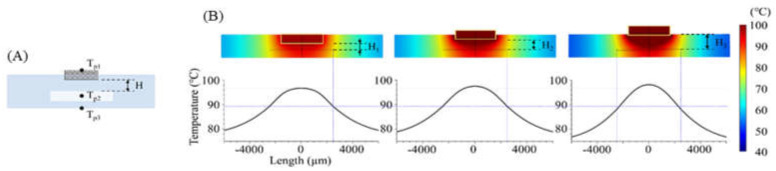
(**A**) Cross sectional view of the microchip (across A-A’ in Figure 1). While changing the distance between the chamber and the heater, the temperature on three points was calculated using FEM simulation (T_p1_: heater, T_p2_: PCR sample, T_p3_: microchip surface). (**B**) Simulated temperature profile of the microchip when H_1_, H_2_, and H_3_ were 600, 950, and 1300 μm, respectively.

The temperature distribution over the entire PCR microchip was obtained via FEM simulation. As shown in Figure 5, heat transfer from the heater was analyzed in three instances: for a distance between the chip resistor heater and the reaction chamber (H) of 600 μm (H = H_1_ = 600 μm), when the heater was inserted half into the microchip (H = H_2_ = 950 μm), and when the heater was placed on the microchip (H = H_3_ = 1300 μm). Figure 5B shows the temperature distribution on the microchip when the temperature of the sample in the chamber was heated to 94 °C. Additionally, a distance-temperature graph was obtained from the temperature data for line A-A’ of Figure 1 that passed through the chamber’s center, showing a temperature variation within 2.3 °C, 2.6 °C, and 3.2 °C when the distance H was H_1_, H_2_, and H_3,_ respectively.

Table 2 displays the heating time when the sample temperature (T_p2_) reached 94 °C, along with the T_p1_ and T_p3_ temperatures at that time. As the distance between the heater and the chamber decreased, less time was needed for T_p2_ to be heated to the target temperature, resulting in an increasingly uniform sample temperature. For a distance H of 600 μm (H_1_), the time to reach the target temperature was 3.6 s and 8.8 s faster than for the distances represented by H_2_ and H_3_, respectively. In addition, the temperature uniformity in the chamber was shown with the standard deviation of the temperature at T_p2_, and the microchip with distance H (600 μm) yielded a more uniform temperature than other conditions. The simulation results demonstrated the heater’s capability with distance H = 600 μm when used for controlling the thermocycling temperature with the temperature sensor and the MCU.

A glass microchip with a 3D structure was successfully executed using SLE. The heater slot, sample chamber, inlet, and outlet structures were etched on a single glass sheet as designed.

**Figure 6:**
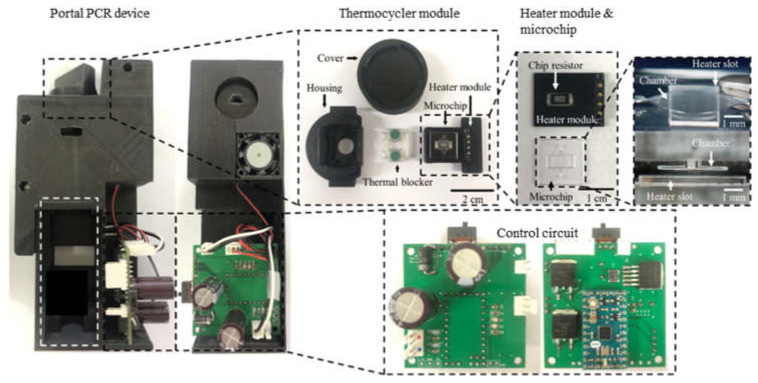
The fabricated PCR device. It consists of the thermocycler module and the control circuit with the MCU. The 3D glass microchip was successfully manufactured by the SLE process. The housing for enclosing all components was fabricated using a 3D printer.

Next, the fabricated heater module on a PCB was integrated with the microchip, as shown in Figure 6. The entire housing and cover were printed with black PLA filament using an FDM-type 3D printer. However, the printed part around the heater module was deformed after several thermo cycling tests due to its low glass transition temperature of around 60 °C to 65 °C. Consequently, an additional housing part that served as a thermal blocker was printed using polycarbonate (PC) filament. The resulting glass transition temperature was about 147 °C; therefore, the PC thermal blocker was not deformed while repeating thermal cycling.

The thermocycling control function was performed by connecting the thermocycler module to the control circuit with the MCU. Using the PID control scheme required obtaining the current temperature of the sample. However, placing a temperature sensor in the sample chamber that can only hold a sample comprising a few micro-liters is a challenging prospect. In addition, while sealing the inlet and the chamber’s outlet is difficult, an incomplete seal may result in the evaporation of the sample. Because of the inability to directly measure the current temperature of the sample, the heater’s temperature was monitored via its associated thermistor, and the temperature of the sample was calculated based on the simulation result of the relationship between the heater temperature and the sample temperature.

**Figure 7:**
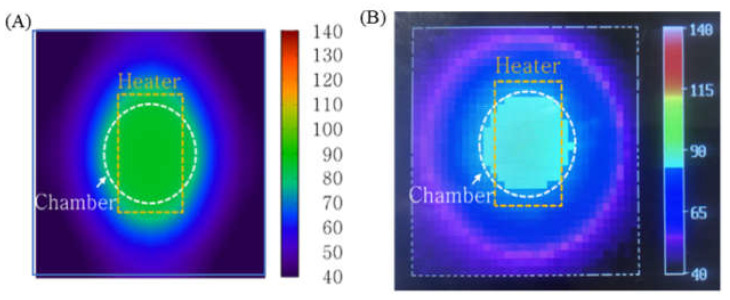
(**A**) Simulation result visualizing the temperature distribution on the surface of the microchip (**B**) Thermal image of the microchip surface using a thermal imaging camera. The temperature profile of the sample inside the chamber was uniform.

To characterize the performance of the PID temperature control, the temperature distribution of the microchip was simulated and measured (Figure 7). For this measurement, a thermal imaging camera was used. While the thermal image does not show the temperature of the sample inside the microchip, it does provide the temperature distribution on the surface of the microchip. Figure 7A illustrates that the heat transferred from the chip resistor heater to the outside of the microchip and the temperature on the surface of the microchip (above the chamber) was maintained at 92 °C. In the thermal image shown in Figure 7B, the microchip’s surface temperature distribution was similar to the shape seen in Figure 7A.

**Figure 8:**
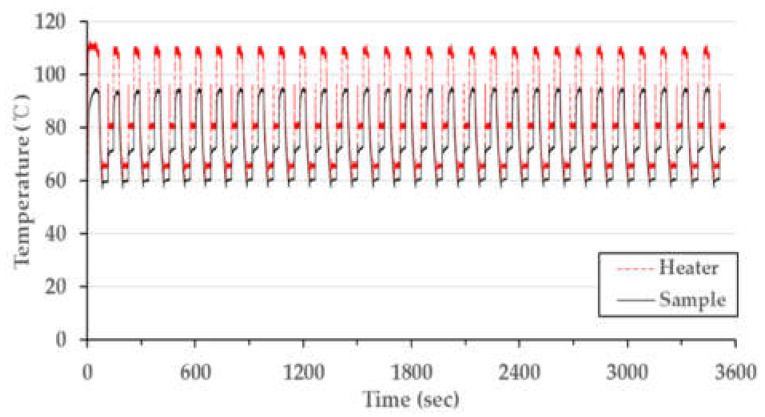
Measured PCR thermocycling profile with PID control.

After characterizing the temperature distribution of the microchip, the PCR thermocycling experiment was conducted. This procedure involved operating 30 cycles with the condition matching the conventional PCR condition shown in Table 3 (a single cycle: 30 s at denaturation, 30 s at annealing, and 30 s at extension, including a 30 s pre-denaturation step at 95 °C). Figure 8 shows the temperature data as a time and temperature graph. The fabricated chip resistor heater was well-controlled to the target temperature, and the thermocycler demonstrated constant performance for 30 cycles. The total PCR thermocycling process consumed 3511 s, which can be broken down to the duration of each part of the procedure as follows: the steps for the 30 cycles comprised 77% (2730 s) of the time taken, while the pre-denaturation step took 1% (30 s) of the time, and 21% (751 s) represents the time needed for heating and cooling between each step. The heating rate from the extension step to the denaturation step was 28.8 °C/s; meanwhile, the heating rate from the annealing step to the extension step was 15.0 °C/s, and the cooling rate was 1.4 °C/s. The reason for the faster heating rate from extension to denaturation compared to that from annealing to the extension has not yet been determined. We assume that the air around the microchip during the extension step was hotter than at annealing, potentially affecting the heating rate. Table 4 compares the performance of our thermocycling device with the reported results for other PCR systems and a commercial PCR machine. The heating rate of our device (21.9 °C/s) was the average value of 28.8 °C/s and 15.0 °C/s. This result was 4 to 8 times faster than those reported in other studies, except for an experiment that used a cement resistor. The rapid heating rate of our device was due to the minimal distance between the chip resistor and the chamber. Our device’s distance of 600 µm was shorter than the distance reported for other PCR systems. Increasing the distance from 600 µm to 1300 µm results in a heating rate that is 2.4 times smaller (according to Table 2). However, the cooling rate was similar to other studies. The air-cooling method in our device did not provide a great effect on discharging heat from the microchip inside the thermocycler housing.

**Figure 9:**
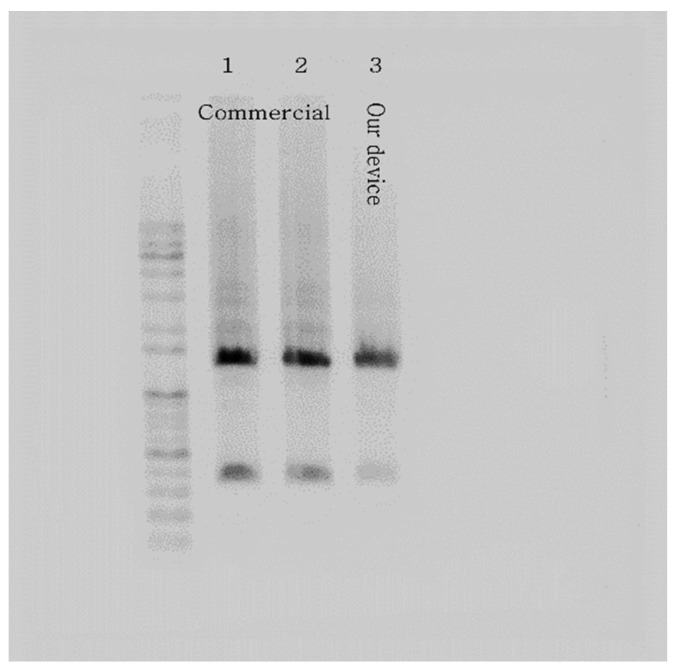
Gel electrophoresis results verify the PCR device with the microchip heated by the chip resistor. The picture shows strong bands of ZFP36 DNA amplified using a commercial PCR machine (samples 1 and 2) and our PCR device (sample 3).

Following the thermocycling experiment, we verified the DNA amplification of our PCR device with the templated DNA (ZFP36). After loading the PCR mix into the microchip and a PCR tube using a pipette, we placed the samples in our PCR device and a commercial PCR machine, respectively. The resulting amplicons from the microchip and the PCR tube were extracted and subjected to conventional gel electrophoresis throughout the experiment. The outcome showed strong bands, validating the performance of our PCR device using the microchip with the chip resistor as a heater. Figure 9 illustrates the gel electrophoresis results, including gel bands of amplified DNA, presenting a comparison of the performance of a commercial PCR machine and our PCR device. In addition, the total PCR time of the commercial PCR was about 74 min, while our PCR device took about 59 min (20% faster).

## 4. Conclusions

We proposed a rapid and inexpensive thermocycler that enables rapid heating of samples using a thin glass chip and a cheap chip resistor for a portable microchip-based PCR device. The simulation of the temperature distribution based on the distance between the reaction chamber and the heater suggested that a smaller distance improved the device’s heating performance. Fabricating a glass microchip using the SLE simplified the prototyping process into two steps, laser processing and etching, thereby reducing the time needed to fabricate the microchip as designed. A small SMD-type chip resistor heater and a thermistor were soldered onto a single PCB (heater module), which was then placed on the microchip. The slot on the microchip for the placement of the chip resistor allowed the easy assembly of the heater module. A PCR device was fabricated by combining a blower fan for cooling, a switching circuit with an MCU (control circuit) to control the heater module, and the thermocycler, which included the heater module. This device demonstrated rapid heating performance with an average heating rate of 21.9 °C/s by locally heating only the chamber area instead of the entire microchip. The proposed formation successfully demonstrated a well-controlled thermocycle and DNA amplification. Moreover, the proposed device’s total PCR time for 30 thermocycles was 20% faster than using a commercial PCR machine. Therefore, the thermocycle technique using a chip resistor can be integrated with a rapid and portable PCR or real-time PCR device for on-site testing.

## Figures and Tables

**Table 1:** Composition ratio of sample for PCR.

Step	Name	Volume (2.0 μL)	Remarks
1	Deionized Water	1.2 μL	
2	DNA template	0.1 μL	TTP (80 μL/mL)
3	Forward Primer	0.1 μL	10 pmol/μL (XL39)
4	Reverse Primer	0.1 μL	10 pmol/μ (VP1.5)
5	5× reaction Buffer	0.4 μL	5× reaction Mix
6	Enzyme	0.1 μL	Premium-Pfu

**Table 2:** Simulation result to time to reach sample temperature of 94 °C for each condition.

Distance between Heater and Chamber	Time to Be Heated to 94 °C at T_p2_	Temperature at Heater Surface (T_p1_)	Temperature of Sample in Chamber (T_p2_)	Temperature of Surface (T_p3_)
H_1_: 600 μm	6.4 s	99.4 °C	94.0 ± 2.3 °C	92.0 °C
H_2_: 950 μm	11.0 s	104.4 °C	94.0 ± 2.6 °C	91.8 °C
H_3_: 1300 μm	15.2 s	111.4 °C	94.0 ± 3.2 °C	91.5 °C

**Table 3:** Thermocycle conditions and heating/cooling rate.

	Thermocycle Step Changing	Transition Time	Transition Rate	Thermocycle Step	Step Time
Start	① 25 ℃→95 ℃	3.2 s	21.8 ℃/s	② 95 ℃ (Pre-Denaturation)	30.0 s
30 cycles	① 72 ℃→95 ℃	0.8 s	28.8 ℃/s	② 95 ℃ (Denaturation)	30.0 s
	③ 95 ℃→60 ℃	24.5 s	−1.4 ℃/s	④ 60 ℃ (Annealing)	30.0 s
	⑤ 60 ℃→72 ℃	0.8 s	15.0 ℃/s	⑥ 72 ℃ (Extension)	30.0 s
Total time	3511 s (58.5 min)				

**Table 4:** Comparison of thermocycle type, size, and speed.

	PCR Type	PCR Chip	Heater Type	Thermocycler Size (mm^3^)	Heating Rate (°C/s)	Cooling Rate (°C/s)
Our device	Conventional	Glass	Chip resistor	40 × 40 × 25	21.9 (averaged)	1.4
Wong et al. [8]	Conventional	N/A	Thermos	N/A	3.5	1.9
Lim et al. [16]	Real-time	Thin film	Pt	50 × 30 × 20	2.9	1.3
Kaprou et al. [20]	Conventional	PMMA	Cu (PCB)	depending on the PCR chip size	1.4	0.6
Gou et al. [21]	Digital	PDMS	Peltier	90 × 90 × 50	5.0	4.0
M.G. et al. [23]	Real-time	PMMA	Cement resistor	N/A	0.5	1.4
Zhu et al. [33]	Conventional	Graphene	Graphene	N/A	4.8	9.3
Commercial PCR (TaKaRa)	Conventional	PCR tube	Peltier	96 well tube plate	3.0	2.0

## Data Availability

Not applicable.
